# Tirzepatide and the gut microbiota–obesity axis: metabolic mechanisms and therapeutic perspectives

**DOI:** 10.3389/fmicb.2026.1901528

**Published:** 2026-07-10

**Authors:** Santiago Cadena-Ullauri, Andrés S. Cadena Paredes, Lenín Guamán-Herrera, Jhon Imbaquingo-Espinales, Viviana A. Ruiz-Pozo, Rafael Tamayo-Trujillo, Patricia Guevara-Ramírez, Elius Paz-Cruz, Ana Karina Zambrano

**Affiliations:** Universidad UTE, Facultad de Ciencias de la Salud Eugenio Espejo, Centro de Investigación Genética y Genómica, Quito, Ecuador

**Keywords:** dysbiosis, gut–brain axis, healthcare, incretin therapy, microbial metabolites

## Abstract

Obesity is a chronic multifactorial metabolic disorder characterized by adipose tissue dysfunction, insulin resistance, low-grade inflammation, and altered energy homeostasis. Evidence indicates that the gut microbiota contributes to obesity-related metabolic dysfunction through microbial metabolite production, intestinal barrier regulation, immune activation, bile acid signaling, and gut–brain communication. This narrative review, supported by a structured literature search, synthesizes current evidence on the gut microbiota–obesity axis and the potential role of tirzepatide in modulating microbiota-related metabolic pathways. Microbiota-derived metabolites, including short-chain fatty acids, bile acids, tryptophan derivatives, trimethylamine N-oxide, and fatty acid-derived compounds, may exert beneficial, detrimental, or context-dependent effects depending on microbial composition, diet, host metabolic status, and tissue-specific signaling pathways. Lifestyle, dietary patterns, host genetics, and medication exposure can further modulate gut microbiota composition and function, influencing metabolic outcomes. Tirzepatide, a dual glucose-dependent insulinotropic polypeptide receptor and glucagon-like peptide-1 receptor agonist, improves glycemic control and promotes weight loss. Evidence suggests that tirzepatide may also be associated with changes in gut microbiota composition, microbial metabolites, intestinal barrier integrity, and inflammatory signaling. However, whether these microbial changes represent direct pharmacological effects or secondary consequences of weight loss, reduced caloric intake, delayed gastric emptying, improved glucose control, or dietary modification remains unclear. This review summarizes current evidence on the gut microbiota–obesity axis and the potential relevance of tirzepatide-associated microbiota modulation.

## 1 Introduction

Obesity is a chronic, multifactorial, and progressive disease associated with type 2 diabetes mellitus, hypertension, cardiovascular disease, and several cancers (Chakaroun et al., 2026; Phelps et al., 2024; Tamayo-Trujillo et al., 2024). The World Obesity Federation Atlas projects that by 2030, the number of people with obesity worldwide will increase by more than 115% compared to 2010, rising from 524 million to 1.13 billion (Powis et al., 2025). Although body mass index (BMI) remains widely used as a diagnostic parameter, current approaches increasingly recognize obesity as a heterogeneous metabolic disorder shaped by adipose tissue dysfunction, circulating metabolites, dietary patterns, host genetics, pharmacological exposure, and gut microbiota composition (Chakaroun et al., 2026; Mouawad et al., 2025). At the metabolic level, obesity is characterized by adipose tissue expansion, insulin resistance, hyperglycemia, and chronic low-grade inflammation (Vetrani et al., 2022; Cavallari and Schertzer, 2017). Dysregulated adipose tissue hypertrophy alters the abundance and distribution of immune cells within metabolic tissues, contributing to cellular senescence, endothelial dysfunction, secondary tissue damage, and fibrosis. These processes create a pro-inflammatory environment that further impairs insulin sensitivity, energy homeostasis, and cardiometabolic health (Vetrani et al., 2022; Cavallari and Schertzer, 2017; van de Vyver, 2023).

Incretin-based therapies are increasingly being used as part of a pharmacological approach for obesity and obesity-associated metabolic disorders (Caruso et al., 2024; Radparvar and Agrawal, 2025; Lee et al., 2025). These interventions have been shown to improve glycemic control and body weight by enhancing glucose-dependent insulin secretion, decreasing glucagon secretion, delaying gastric emptying, reducing appetite, and modulating central pathways involved in satiety and energy intake (Caruso et al., 2024; Radparvar and Agrawal, 2025; Nauck et al., 2026). The effects target key components of metabolic syndrome, including hyperglycemia, insulin resistance, excess adiposity, and cardiometabolic risk (Caruso et al., 2024; Radparvar and Agrawal, 2025; Ngabea and Dimeji, 2025). However, the metabolic benefits of incretin-based therapies may extend beyond classical endocrine mechanisms, as changes in nutrient flow, gastrointestinal motility, bile acid metabolism, inflammation, and dietary intake may also interact with gut microbial ecology (Wang et al., 2025; Madsen et al., 2019; Ma et al., 2025).

In this context, the gut microbiota has emerged as an important regulator of energy metabolism, immune signaling, intestinal barrier integrity, and host–microbe communication (El-Sayed et al., 2021; Longo et al., 2023; Lin et al., 2022; Bakker and Nieuwdorp, 2017). Gut microorganisms contribute to nutrient extraction and generate bioactive metabolites that interact with host receptors and metabolic pathways (Lin et al., 2022; Bakker and Nieuwdorp, 2017; Sehgal and Khanna, 2021). These include short-chain fatty acids (SCFAs), bile acid derivatives, tryptophan-derived indoles, trimethylamine N-oxide (TMAO), and amino acid- or fatty acid-derived compounds (Lin et al., 2022). Depending on microbial composition, diet, host metabolic status, and tissue-specific signaling, these metabolites may exert beneficial, detrimental, or context-dependent effects on obesity-related metabolic dysfunction (Lin et al., 2022; Bakker and Nieuwdorp, 2017; Sehgal and Khanna, 2021). For instance, SCFAs can regulate intestinal barrier integrity, enteroendocrine hormone secretion, appetite, and glucose metabolism. Propionate and butyrate have also been implicated in cholesterol metabolism and inflammatory pathway modulation through effects on lipoprotein-related processes (Lin et al., 2022; Kravetz et al., 2020; Flint et al., 2014; Chang et al., 2025). In addition, microbial metabolites including glutamate, palmitate, and elaidate may compromise intestinal barrier integrity, facilitating the translocation of pro-inflammatory molecules such as lipopolysaccharide (LPS), thereby promoting host inflammatory responses (Zwartjes et al., 2021; Yun et al., 2020; Takeuchi et al., 2023). Furthermore, the microbiota synthesizes a variety of tryptophan-derived metabolites that may regulate intestinal barrier function, immune signaling, and mucosal homeostasis in a context-dependent manner (Du et al., 2024). Similarly, bile acid derivatives modulate FXR and TGR5 signaling pathways involved in lipid metabolism, insulin sensitivity, energy expenditure, and GLP-1 secretion (Wahlström et al., 2016; Geng et al., 2022). Other microbial products may influence metabolic endotoxemia, immune activation, adipose tissue inflammation, gut–brain communication, and circadian regulation (Lin et al., 2022; Dimeji and Ayodeji, 2025). Together, these mechanisms suggest that the gut microbiota–obesity axis is not limited to nutrient extraction or inflammation, but also involves endocrine, immune, metabolic, and neural pathways that affect energy intake, fat storage, and metabolic homeostasis (Longo et al., 2023; Cryan, 2019; Van Son et al., 2021).

Tirzepatide, a dual glucose-dependent insulinotropic polypeptide (GIP) receptor and glucagon-like peptide-1 (GLP-1) receptor agonist, has demonstrated efficacy in improving glycemic control and promoting weight loss in individuals with obesity and metabolic disease (Baker et al., 2024; Wang et al., 2025). Compared with other obesity-approved pharmacological therapies, tirzepatide has shown efficacy in body weight reduction and may represent a cost-effective therapeutic option for obesity management, although tolerability, accessibility, and long-term safety remain important considerations (Liu et al., 2025; Lafferty et al., 2023). Tirzepatide improves metabolic syndrome-related abnormalities through glucose-dependent stimulation of insulin secretion, modulation of glucagon dynamics, delayed gastric emptying, appetite suppression, reduced energy intake, and improvements in body weight and adiposity (Thiriveedi, 2025; Hossain et al., 2025; Rochira et al., 2024). Beyond its incretin-mediated effects, evidence suggests that tirzepatide may also influence gut microbiota composition and function (Wang et al., 2025; Madsen et al., 2019; Ma et al., 2025; Trapanese et al., 2025). However, it remains unclear whether these microbiota changes represent direct pharmacological effects or secondary consequences of weight loss, reduced caloric intake, improved glycemic control, altered bile acid metabolism, or changes in dietary behavior.

This review aims to present and discuss current evidence on the gut microbiota–obesity axis, with emphasis on microbial taxa, microbiota-derived metabolites, inflammatory and endocrine pathways, and host-related modulators such as diet, lifestyle, genetics, and medication exposure. In addition, it evaluates the potential relationship between tirzepatide and gut microbiota modulation, highlighting whether tirzepatide-associated microbial changes may contribute to metabolic improvement, therapeutic response, and future microbiota-targeted strategies in obesity management.

## 2 Methods

The present article is a narrative review supported by a structured literature search aimed at synthesizing current evidence on the potential role of tirzepatide in modulating the gut microbiota-obesity axis. A structured literature search was conducted using PubMed/MEDLINE, Scopus, Web of Science, and Google Scholar. The search included combinations of the following terms: “tirzepatide,” “GIP receptor agonist,” “GLP-1 receptor agonist,” “gut microbiota,” “obesity,” “metabolites,” “inflammation,” and “host metabolism.” Additional relevant articles were identified through manual screening of reference lists from selected publications.

Eligible publications included peer-reviewed original studies, preclinical studies, clinical trials, observational studies, systematic reviews, meta-analyses, and mechanistic reviews addressing the gut microbiota, obesity, host metabolism, incretin-based therapies, GLP-1 receptor agonists, GIP/GLP-1 receptor agonists, or tirzepatide. Studies not directly related to gut microbiota, obesity, metabolic regulation, or incretin-based therapies were excluded. Studies were selected based on thematic relevance, mechanistic contribution, methodological quality, and relevance to the gut microbiota–obesity–tirzepatide axis.

## 3 Mechanistic insights into gut microbiota and host metabolism interactions

The human gut microbiota participates in a dynamic symbiotic relationship between the host and the intestinal ecosystem. Host-related factors such as diet, age, lifestyle, medication use, and other environmental exposures can shape microbial diversity, composition, and function. In turn, gut microorganisms can influence several physiological processes involved in health and disease (Afzaal et al., 2022; Simões et al., 2021). Gut microorganisms contribute to host metabolism through multiple mechanisms, including digestion of otherwise indigestible dietary components, energy extraction, immune system modulation, resistance to pathogen colonization, and the biosynthesis or transformation of amino acids, vitamins, lipids, and other bioactive metabolites with intestinal and extra-intestinal effects (Lin et al., 2022; Afzaal et al., 2022; Shan et al., 2022; Hou et al., 2022; Bouskra et al., 2008). Therefore, in obesity, the gut microbiota should be understood as a multifactorial complex involving host genetics, diet, lifestyle, bile acid metabolism, immune signaling, endocrine regulation, adipose tissue function, and energy homeostasis, and dysbiosis may contribute to obesity-associated alterations (Turnbaugh et al., 2006).

The gut microbiota is mainly composed of anaerobic microorganisms, reflecting the low-oxygen environment of the colon. Although its composition varies considerably among individuals, the dominant bacterial phyla commonly include Firmicutes, Bacteroidetes, Actinobacteria, Proteobacteria, and Verrucomicrobia (De Vos et al., 2022; Li et al., 2014; Pan et al., 2024; Zhang et al., 2024). Some studies have suggested that obesity could be associated with an increased Firmicutes/Bacteroidetes ratio; however, subsequent studies have reported inconsistent findings, indicating that this ratio is not a universal biomarker of obesity (Lin et al., 2022; Koliada et al., 2017; Sasidharan, 2026). In contrast, reduced microbial diversity and a lower abundance of butyrate-producing bacteria have been more consistently associated with obesity-related metabolic impairment in many studies (Sasidharan, 2026). Therefore, current evidence suggests that obesity-related dysbiosis should not be interpreted solely in terms of broad taxonomic shifts. Instead, it may be more informative to consider changes in microbial diversity, relative taxonomic composition, and functional capacity.

### 3.1 Microbial energy harvest and substrate metabolism

One major mechanism linking the gut microbiota to obesity involves the transformation of dietary and host-derived substrates into bioactive metabolites. Non-digestible carbohydrates, including complex polysaccharides and resistant starches, are fermented by colonic bacteria, supporting microbial growth and generating metabolites that influence host energy balance (Geng et al., 2022; Ruiz-Pozo et al., 2024; Tremaroli and Bäckhed, 2012). This microbial contribution to energy extraction may be particularly relevant in obesity, where altered microbial functional capacity can affect nutrient availability, lipid storage, glucose metabolism, and inflammatory tone (Cavallari and Schertzer, 2017; Bakker and Nieuwdorp, 2017; Enache et al., 2024). SCFAs, mainly acetate, propionate, and butyrate, are produced through the bacterial fermentation of non-digestible dietary carbohydrates (Zambrano et al., 2024a; Ruiz-Pozo et al., 2024). These metabolites act locally in the intestine and systemically after absorption. Butyrate serves as an important energy source for colonocytes and contributes to epithelial barrier integrity, whereas acetate and propionate may participate in hepatic and peripheral metabolic pathways (Zambrano et al., 2024a; Cadena-Ullauri et al., 2025; Xiong et al., 2022).

**Figure 1:**
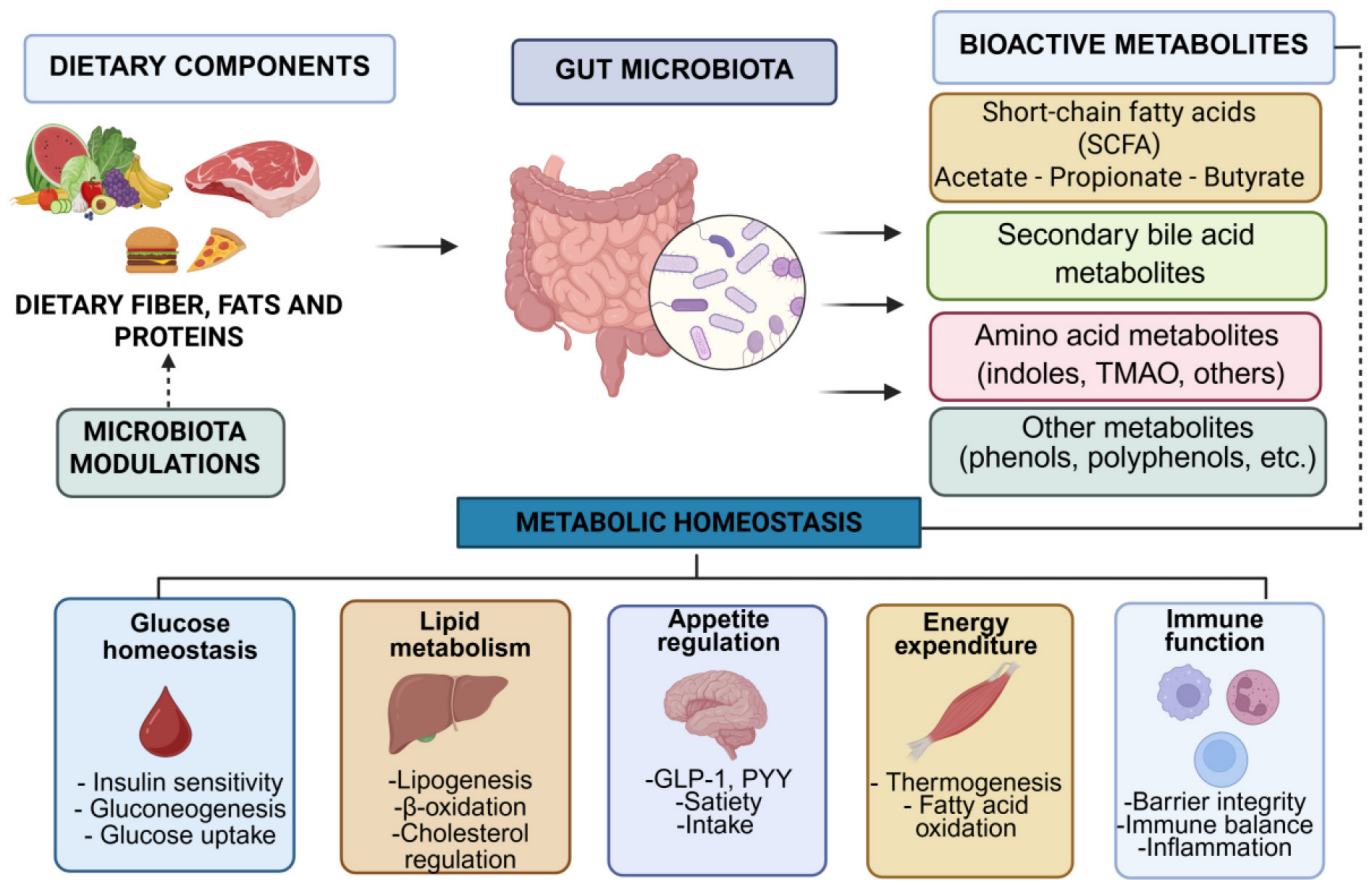
Network of interactions between the gut microbiota and the host's metabolism. The gut microbiota metabolizes dietary components, including fiber, fats, and proteins, into bioactive compounds such as short-chain fatty acids, bile acid derivatives, and amino acid metabolites. These metabolites regulate glucose and lipid metabolism, appetite, energy expenditure, immune function, and overall metabolic homeostasis. Created in BioRender. Cadena-Paredes, A. (2026) https://BioRender.com/hhiul1o.

Although SCFAs have been primarily characterized as products of microbial fermentation, obesity-associated metabolic dysfunction cannot be explained solely by SCFA production (Faradilah et al., 2025; Molan et al., 2025). Gut microorganisms also participate in bile acid transformation, tryptophan metabolism, indole production, amino acid metabolism, and fatty acid modification, generating signaling molecules that may affect intestinal barrier integrity, hepatic metabolism, adipose tissue physiology, insulin sensitivity, and immune regulation (Tremaroli and Bäckhed, 2012; Ruiz-Pozo et al., 2024; Geng et al., 2022; Portincasa et al., 2022; Su et al., 2022). Several microbiota-derived metabolites have been implicated in obesity-related metabolic dysfunction. A summary of selected metabolites and their proposed effects is presented in Tables 1-3; these are divided into *in vitro* studies, animal models, and human studies, and Figure 1 represents the interaction of gut microbiota and the host's metabolism.

**Table 1:** Studies *in vitro* evaluating gut microbiota-derived metabolites associated with obesity.

Study description	Model	Key microbial taxa	Metabolic effect	Biological implications	References
Microbiota metabolites involved in obesity	*In vitro*	*Escherichia coli* and *Salmonella typhimurium*	Delta-valerobetaine → fatty acid oxidation and carnitine ↓	Delta-valerobetaine may impair fatty acid oxidation and carnitine metabolism, potentially favoring lipid accumulation.	Lin et al., 2022; Liu et al., 2021
Overproduction of fatty acids present in obesity by the intestinal microbiota.	Human, mouse, and *in vitro*	*Fusimonas intestine, Marvinbryantia formatexigens*	Elaidate ↑, Palmitate ↑	Elaidate may reduce ZO-1, occludin, and JAM-1 expression, compromising epithelial barrier integrity. Palmitate can activate TLR4/NF-κB inflammatory signaling in adipose tissue and has been associated with insulin resistance.	Takeuchi et al., 2023
Tryptophan, metabolized by the gut microbiota, prevents insulin resistance	Human, mouse models, and *in vitro*	*Burkholderia cenocepacia, Burkholderia pyrrocinia*	Tryptophan → 5-hydroxyindole-3-acetic acid (5-HIAA) ↑	5-HIAA may activate AhR signaling and stimulate TSC2 expression, thereby inhibiting mTORC1-related pathways involved in lipid accumulation and insulin resistance.	Du et al., 2024
		*Escherichia coli, Clostridium* spp, *Bacteroides* spp.	Tryptophan → Indole and Indole-3-Acetic Acid (IAA) ↑	Indole-3-acetic acid may activate AhR signaling and contribute to intestinal mucosal homeostasis, potentially reducing intestinal permeability.	

↑, increased; → , conversion or metabolic pathway; ↓, decreased.

**Table 2:** Animal studies evaluating gut microbiota-derived metabolites associated with obesity.

Study description	Model	Key microbial taxa	Metabolic effect	Biological implications	References
Microbiota metabolites involved in obesity	Animals (Mice)	*Escherichia coli, Klebsiella* spp. and *Citrobacter* spp.	Carnitine/choline → TMAO (Trimethylamine N-Oxide) ↑	TMAO has been associated with intestinal inflammation, visceral fat accumulation, and insulin resistance-related metabolic alterations.	Wu et al., 2019
Multi-omics analysis of the role of the gut microbiota in the development of obesity	Mouse models	*Anaerotruncus colihominis*	Glutamate → ZO-1 ↓ and Occludin ↓	Glutamate-associated changes may reduce intestinal barrier proteins such as ZO-1 and occludin, increasing intestinal permeability and contributing to obesity-associated metabolic dysfunction.	Chang et al., 2025
Metabolites produced by gut microbiota that are related to obesity	Mouse models	*Clostridium tyrobutyricum*	Butyrate ↑	Butyrate may promote *ANGPTL4* expression, which inhibits lipoprotein lipase and may influence lipid storage.	Zwartjes et al., 2021; Yun et al., 2020; Korecka et al., 2013

↑, increased; → , conversion or metabolic pathway; ↓, decreased.

**Table 3:** Human studies evaluating gut microbiota-derived metabolites associated with obesity.

Study description	Model	Key microbial taxa	Metabolic effect	Biological implications	References
Microbiota metabolites involved in obesity	Human	Bacteroidetes, Firmicutes, Veillonellaceae, *Coprococcus catus*, Lachnospiraceae	SCFAs (propionate) ↑	Propionate can be produced through succinate, acrylate, and propanediol pathways. It may serve as an energy substrate for colonocytes and influence cholesterol synthesis.	Flint et al., 2014; Lin et al., 2022; Reichardt et al., 2014
Meta-analysis of metabolites produced by the gut microbiota and their relationship with obesity	*In silico* (humans)	*Lactobacillus paracasei JS1*	Isoflavones → equol → IL6 ↓	Equol may reduce IL-6-mediated inflammatory signaling, a pathway associated with insulin resistance and free fatty acid production.	Oh et al., 2022
Overproduction of fatty acids present in obesity by the intestinal microbiota.	Human	*Fusimonas intestine*	*fadR genetic regulator* ↑	Consuming fats causes this regulator to promote the expression of the accB, accC, and fabK genes, which are involved in fatty acid synthesis and have a positive correlation with body mass index and elevated glucose levels in obese individuals.	Takeuchi et al., 2023

↑, increased; → , conversion or metabolic pathway; ↓, decreased.

### 3.2 Microbiota-derived metabolites as mediators of obesity-related dysfunction

Microbiota-derived metabolites can exert beneficial, detrimental, or context-dependent effects depending on microbial composition, diet, host metabolic status, and tissue-specific signaling pathways (Geng et al., 2022; Agus et al., 2021; Canfora et al., 2019; Liu et al., 2022). For example, propionate-producing bacteria, including members of Bacteroidetes, Firmicutes, Veillonellaceae, *Coprococcus catus*, and Lachnospiraceae, may influence host energy metabolism and cholesterol synthesis through succinate, acrylate, and propanediol pathways (Flint et al., 2014; Lin et al., 2022; Reichardt et al., 2014). In contrast, other microbial metabolites, such as trimethylamine N-oxide (TMAO), delta-valerobetaine, glutamate, elaidate, and palmitate, have been associated with intestinal inflammation, altered fatty acid oxidation, impaired epithelial barrier integrity, visceral fat accumulation, or insulin resistance in experimental and multi-omics studies (Takeuchi et al., 2023; Lin et al., 2022; Liu et al., 2021).

**Figure 2:**
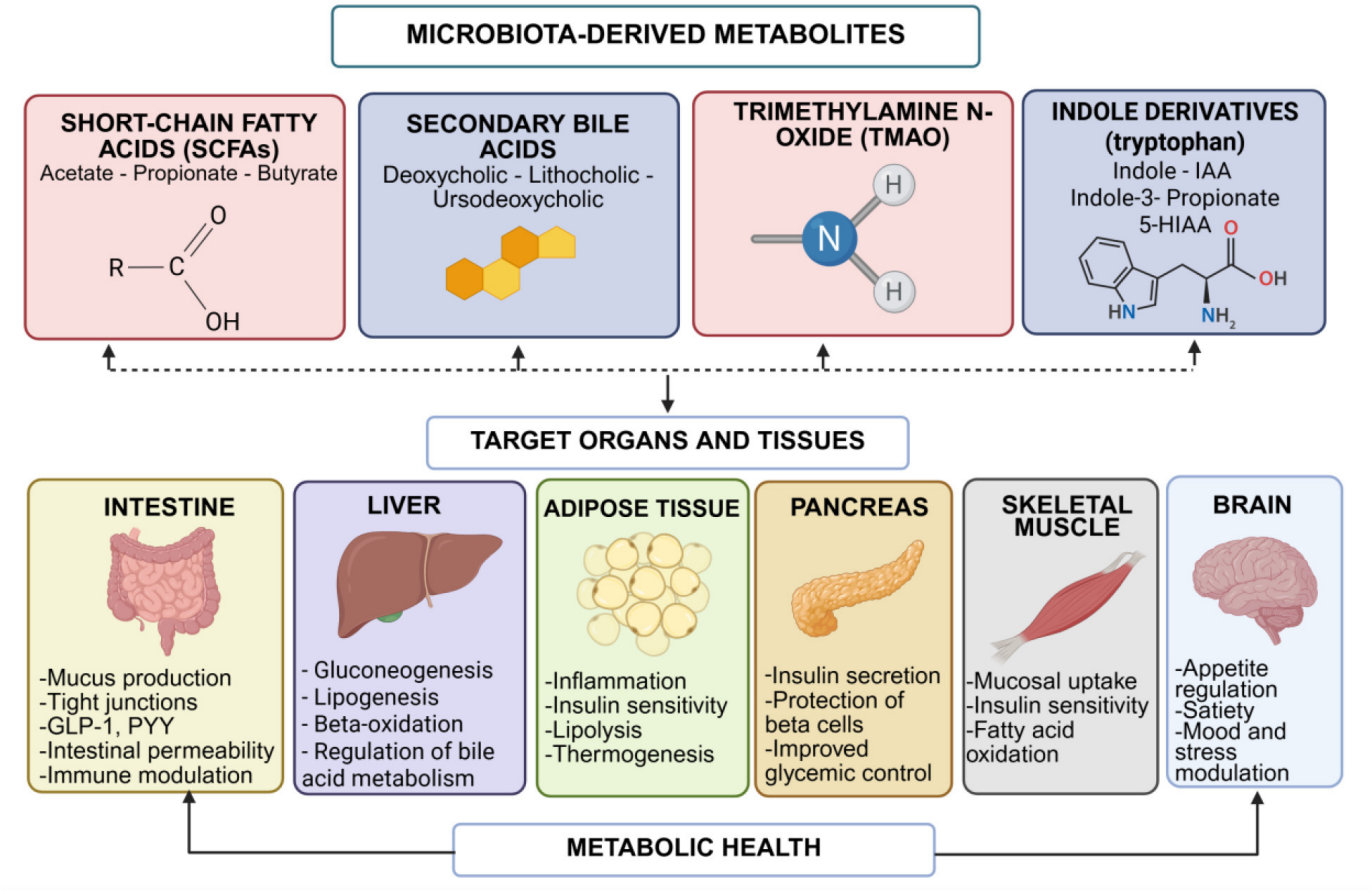
Microbiota-derived metabolites and their metabolic effects. Microbiota-derived metabolites, including short-chain fatty acids, secondary bile acids, trimethylamine N-oxide (TMAO), and indole derivatives, exert systemic effects on multiple organs. Through their actions on the intestine, liver, adipose tissue, pancreas, skeletal muscle, and brain, these metabolites contribute to the regulation of metabolic and immune functions. Created in BioRender. Cadena-Paredes, A. (2026) https://BioRender.com/hhiul1o.

Some microbial metabolites may also exert potentially protective metabolic effects. Butyrate-producing bacteria, including *Oscillospira* and *Clostridium tyrobutyricum*, have been linked to the regulation of lipid storage through mechanisms involving angiopoietin-like 4 and lipoprotein lipase inhibition (Zwartjes et al., 2021; Yun et al., 2020; Korecka et al., 2013). Similarly, microbial metabolism of isoflavones to equol may reduce interleukin-6-mediated inflammatory signaling, while tryptophan-derived metabolites such as 5-hydroxyindole-3-acetic acid may activate aryl hydrocarbon receptor signaling and modulate pathways involved in insulin resistance and lipid accumulation (Oh et al., 2022). However, these effects should be interpreted cautiously because much of the evidence comes from animal models, *in vitro* studies, *in silico* analyses, or integrated multi-omics approaches that require further validation in longitudinal human cohorts. Figure 2 represents the metabolites derived from the microbiota and their importance in the organism.

### 3.3 SCFA-mediated receptor signaling and epigenetic regulation

SCFAs remain central to these mechanisms because they link microbial fermentation with endocrine, immune, and epigenetic regulation. Acetate, propionate, and butyrate can activate G-protein-coupled receptors, including FFAR2 and FFAR3, and may influence intestinal gluconeogenesis, enteroendocrine signaling, and inflammatory pathways (Geng et al., 2022; Portincasa et al., 2022; Mishra et al., 2020; Schlatterer et al., 2021). SCFAs have also been reported to inhibit histone deacetylases, suggesting that microbiota-derived metabolites may participate in epigenetic regulation of host metabolic responses (Geng et al., 2022; Portincasa et al., 2022). These mechanisms are relevant to obesity because altered SCFA signaling may affect insulin sensitivity, adipose tissue inflammation, gut barrier function, and appetite-related endocrine responses (Agus et al., 2021; Liu et al., 2022; Lin et al., 2022).

### 3.4 Bile acid metabolism and host metabolic signaling

Bile acid metabolism represents another important pathway connecting gut microbiota with obesity-related metabolic regulation. The gut microbiota participates in the conversion of primary bile acids into secondary bile acids, which can act as signaling molecules through receptors such as farnesoid X receptor and Takeda G protein-coupled receptor 5 (Xiang et al., 2026; Qi et al., 2024; Zangerolamo et al., 2025). Through these pathways, bile acids may influence lipid metabolism, glucose homeostasis, energy expenditure, inflammation, and enteroendocrine hormone secretion. Because bile acid signaling can influence enteroendocrine function and incretin-related pathways, this mechanism is particularly relevant when considering GLP-1 receptor agonists and dual GIP/GLP-1 receptor agonists (Xiang et al., 2026; Qi et al., 2024; Perino and Schoonjans, 2022; Fuchs and Trauner, 2022). However, the specific contribution of microbiota-derived bile acid changes to obesity and tirzepatide response remains insufficiently defined and should be addressed in future studies.

### 3.5 Tryptophan metabolism, indole derivatives, and AhR signaling

Tryptophan metabolism represents another pathway through which the microbiota may influence obesity-associated physiology. Dietary tryptophan can be metabolized by host and microbial pathways, including the kynurenine pathway, mediated by indoleamine 2,3-dioxygenase and tryptophan 2,3-dioxygenase, and the serotonin pathway, which generates 5-hydroxytryptamine (5-HT), a mediator involved in intestinal motility and gut–brain communication (Geng et al., 2022; Su et al., 2022). Microbial conversion of tryptophan into indole derivatives may also contribute to intestinal barrier regulation and immune signaling, although the direction and magnitude of these effects may vary according to microbial composition and host metabolic status (Geng et al., 2022; Su et al., 2022). Nevertheless, the metabolic consequences of tryptophan-derived metabolites are likely pathway-specific and context-dependent, and their role in obesity should not be interpreted as uniformly protective or detrimental.

### 3.6 Barrier dysfunction, metabolic endotoxemia, and inflammation

Obesity-associated dysbiosis may contribute to chronic low-grade inflammation, often referred to as meta-inflammation. Altered microbial composition and impaired epithelial barrier integrity can increase host exposure to microbial components, including LPS, thereby promoting inflammatory signaling in metabolic tissues (Bander, 2020; Tume et al., 2025; Candelli et al., 2021; Scheithauer et al., 2020).

Gut microbiota can also interact with the immune system, including both innate and adaptive immunity (Hou et al., 2022). For instance, the Secretory Immunoglobulin A (SIgA), a key component of mucosal immunity, helps maintain host–microbe homeostasis by limiting bacterial adhesion to the epithelium, neutralizing pathogens, and shaping the composition of intestinal microbial communities (Pietrzak et al., 2020; Alexander et al., 2014). In consequence, when microbiota balance is disrupted, dysbiosis may compromise epithelial barrier function, favor abnormal microbial expansion, and facilitate inflammatory responses (Hou et al., 2022; Hapfelmeier et al., 2010). Similarly, the adaptive immune system is influenced by microbial signals that contribute to the maturation and regulation of B- and T-cell responses, promoting tolerance toward commensal microorganisms while preserving the capacity to respond to pathogens (Hou et al., 2022; Zhao and Elson, 2018).

These immune–microbial interactions are metabolically relevant because chronic low-grade inflammation is a central feature of obesity and insulin resistance (Bander, 2020; Tume et al., 2025; Candelli et al., 2021). Dysbiosis-related barrier dysfunction can increase exposure to bacterial components, such as LPS, thereby promoting inflammatory signaling in metabolic tissues (Tume et al., 2025; Candelli et al., 2021). This process may contribute to adipose tissue dysfunction, altered lipid storage, impaired insulin sensitivity, and systemic metabolic imbalance (Bander, 2020). Similarly, increased intestinal permeability can facilitate the translocation of LPS from the intestinal lumen into the systemic circulation, a phenomenon known as metabolic endotoxemia that is related to the intake of fats and sugars (Dimeji and Ayodeji, 2025). This condition has been associated with activation of Toll-like receptor 4 (TLR4)-dependent signaling pathways and the subsequent production of pro-inflammatory mediators that contribute to chronic low-grade inflammation (Su et al., 2022). These responses may involve cytokine-mediated pathways that contribute to insulin resistance, adipose tissue dysfunction, altered lipid storage, and type 2 diabetes mellitus (T2DM) risk (Scheithauer et al., 2020). Anti-inflammatory mediators such as interleukin-10 (IL-10) may counteract these processes, and lower IL-10 levels have been reported in individuals with obesity compared with lean controls in some studies (Hong et al., 2009; Esposito et al., 2003). Therefore, the gut microbiota may influence host metabolism not only by regulating nutrient processing and metabolite production, but also by shaping immune response and inflammatory pathways involved in obesity-associated metabolic disease (Bander, 2020; Tume et al., 2025; Candelli et al., 2021). Nevertheless, this association should be interpreted cautiously, as inflammatory profiles in obesity are influenced by adiposity, diet, metabolic status, medication use, and other host-related variables.

### 3.7 Enteroendocrine and gut–brain–microbiota signaling

Microbiota-derived metabolites may also influence appetite and glucose homeostasis through enteroendocrine and neural pathways. SCFAs can also activate free fatty acid receptors, particularly FFAR2 and FFAR3, expressed in intestinal epithelial and enteroendocrine cells. Activation of these receptors promotes the secretion of (GLP-1) and peptide YY (PYY), two hormones involved in appetite regulation, satiety, gastric emptying, insulin secretion, and postprandial glucose homeostasis (Lin et al., 2022; Shan et al., 2022; Islam et al., 2026). Through these pathways, microbial metabolites may influence energy intake, postprandial glucose control, and host metabolic flexibility (Angelini et al., 2024; Rovella et al., 2021; Zhang et al., 2018).

Other appetite- and metabolism-related hormones, including peptide YY, ghrelin, leptin, and insulin, may also interact with microbial signals and participate in the regulation of satiety, hunger, gastric emptying, energy intake, and metabolic flexibility (Angelini et al., 2024; Rovella et al., 2021; Zhang et al., 2018; Brettle et al., 2022; Asadi et al., 2022). These mechanisms converge within the gut–brain–microbiota axis, in which microbial metabolites are sensed by enteroendocrine cells and neural pathways, including vagal afferent signaling, while central and autonomic outputs influence gastrointestinal motility, hormone secretion, intestinal permeability, and the luminal environment (Longo et al., 2023; Cryan, 2019; Asadi et al., 2022; Cani and Knauf, 2016).

**Figure 3:**
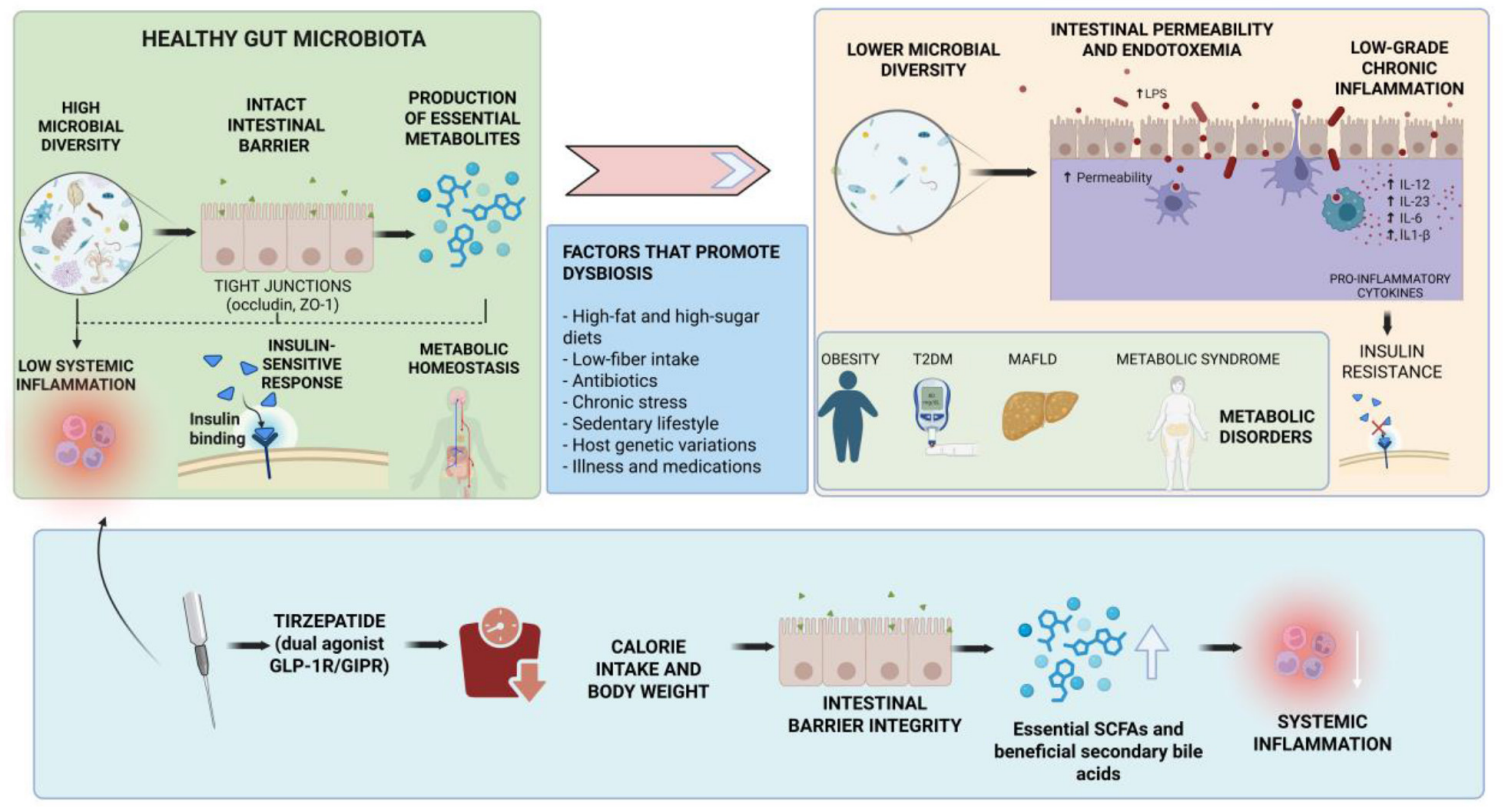
Intestinal dysbiosis, metabolic disorders, and the potential role of tirzepatide. Gut dysbiosis is characterized by reduced microbial diversity, impaired intestinal barrier integrity, increased endotoxemia, and chronic low-grade inflammation, which collectively contribute to metabolic disorders as a type 2 diabetes mellitus (T2DM) and metabolic dysfunction-associated fatty liver disease (MAFLD). Tirzepatide may improve metabolic health by reducing body weight, enhancing intestinal barrier function, promoting beneficial microbial metabolites, and attenuating systemic inflammation. Created in BioRender. Cadena-Paredes, A. (2026) https://BioRender.com/hhiul1o.

In addition to SCFAs, gut microorganisms can produce or modulate neuroactive compounds involved in gut–brain communication. Gamma-aminobutyric acid (GABA), for example, can be produced by specific intestinal bacteria and may influence appetite-related signaling through the gut–brain axis (Shan et al., 2022; Islam et al., 2026). However, this should be interpreted with caution, as the contribution of microbiota-derived GABA to circulating or central nervous system signaling remains context-dependent and is influenced by various factors (Braga et al., 2024; Strandwitz et al., 2018; Icer et al., 2024). Therefore, microbial GABA could be part of a neuroendocrine network connecting the gut microbiota with appetite and energy regulation (Shan et al., 2022; Islam et al., 2026). Figure 3 shows metabolic disorder and intestinal dysbiosis.

### 3.8 Integrated model of microbiota-driven metabolic dysfunction in obesity

Microbiota–obesity interactions are bidirectional and involve multiple interconnected mechanisms, rather than being driven by a single bacterial taxon or metabolite (Longo et al., 2023; Lin et al., 2022; Torres-Fuentes et al., 2017). Microbial fermentation, SCFA signaling, bile acid transformation, tryptophan metabolism, epithelial barrier integrity, metabolic endotoxemia, immune activation, adipose tissue dysfunction, insulin resistance, and gut–brain communication collectively shape host metabolic homeostasis (Longo et al., 2023; Faradilah et al., 2025; Agus et al., 2021; Lin et al., 2022; Perino and Schoonjans, 2022; Fuchs and Trauner, 2022). This network is relevant for evaluating incretin-based therapies, including tirzepatide, because microbiota-associated changes during treatment may reflect direct drug-related effects, secondary consequences of weight loss and improved glycemic control, or broader host–microbiota metabolic adaptation (Trapanese et al., 2025; Angelini et al., 2024; Koufakis et al., 2025).

## 4 Modulators of the gut microbiota in obesity: lifestyle, diet, host genetics, and medication exposure

**Figure 4:**
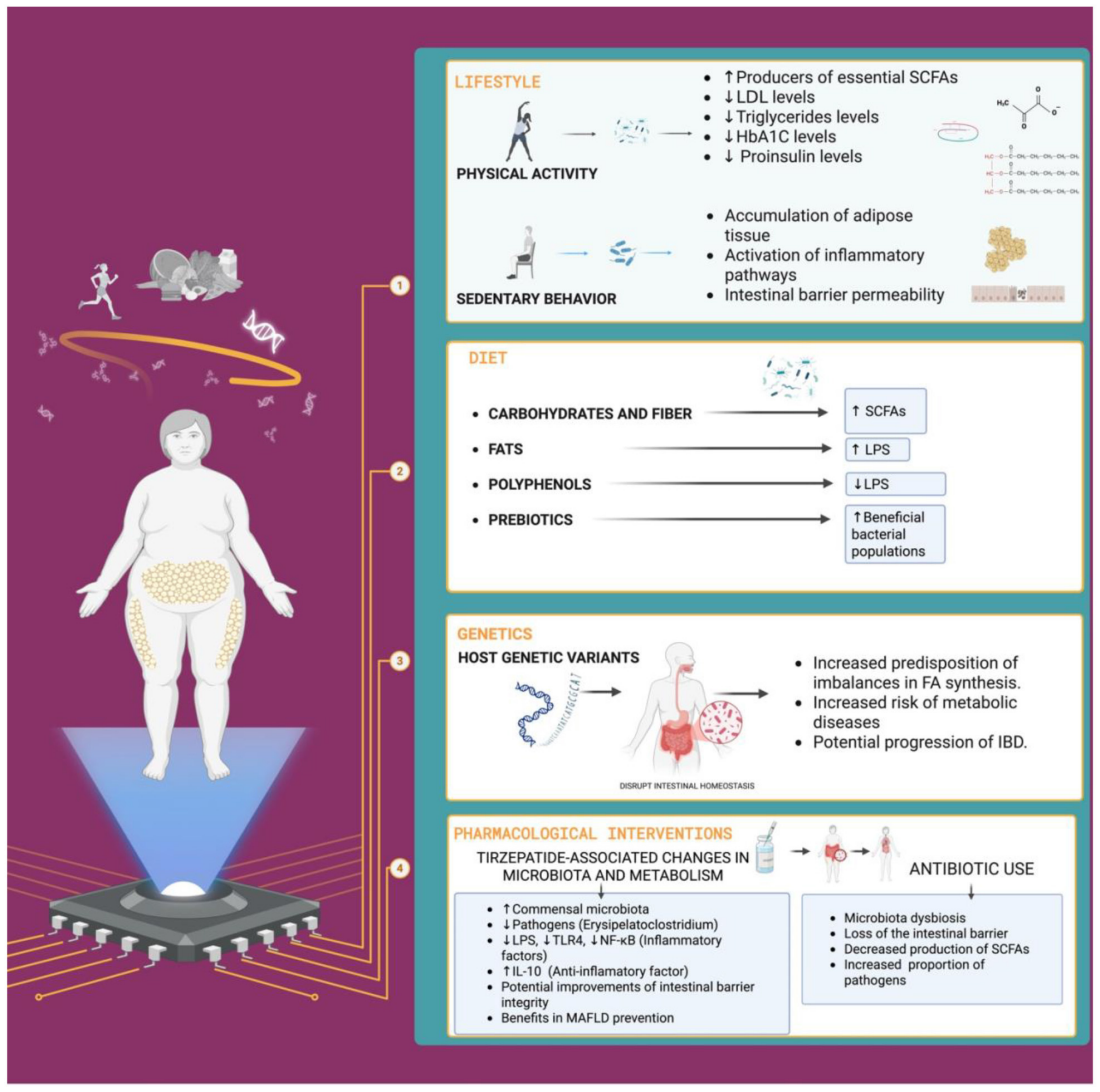
Modulators of the gut microbiota in obesity and their metabolic implications. The figure summarizes key factors that influence gut microbiota composition and function in obesity, including lifestyle, diet, host genetics, and medication exposure. Abbreviations: FA, fatty acid; Hb1Ac, glycated hemoglobin; IBD, inflammatory bowel disease; IL-10, interleukin-10; LDL, low-density lipoprotein; LPS, lipopolysaccharide; MAFLD, metabolic dysfunction-associated fatty liver disease; NF-κB, nuclear factor kappa B; SCFAs, short-chain fatty acids; TLR4, Toll-like receptor 4. Created in BioRender. Cadena-Ullauri, S. (2026) https://BioRender.com/hhiul1o.

The composition and functional capacity of the gut microbiota are shaped by multiple modifiable and non-modifiable factors, including lifestyle, diet, host genetics, medication exposure, and metabolic status (Longo et al., 2023; Cadena-Ullauri et al., 2024; Zambrano et al., 2024b; Cuevas-sierra, 2019). In obesity, these factors may influence microbial diversity, taxonomic abundance, metabolite production, epithelial barrier integrity, and immune–metabolic signaling (Longo et al., 2023; Lin et al., 2022; Sehgal and Khanna, 2021). Figure 4 represents some of the major modulators of the gut microbiota in obesity.

### 4.1 Lifestyle and physical activity

Lifestyle factors, particularly physical activity and sedentary behavior, can influence gut microbiota diversity and composition. Physically active individuals generally show higher microbial diversity and enrichment of taxa associated with metabolic health. In elite athletes, endurance activity has been associated with increased abundance of genera such as *Oscillospira, Bacteroides*, while higher levels of *Faecalibacterium prausnitzii, Bifidobacterium adolescentis*, and *Christensenella*, have been reported in active adults (Strasser et al., 2021; Muralidharan et al., 2021). These microbial patterns may also reflect dietary habits commonly associated with active lifestyles, including higher intake of fiber-rich and nutrient-dense foods.

In contrast, sedentary behavior has been associated with microbial alterations that may favor obesity-related metabolic dysfunction. Some studies have reported increased Firmicutes and reduced Actinobacteria and Proteobacteria in sedentary individuals, while obesity has also been linked to shifts involving Firmicutes and Bacteroidetes (Xu et al., 2023; Ley et al., 2005). Nevertheless, these findings should be interpreted cautiously because broad taxonomic ratios, particularly the Firmicutes/Bacteroidetes ratio, are inconsistent across studies and should not be considered universal biomarkers of obesity.

The influence of lifestyle is particularly relevant when interpreting microbiota changes during tirzepatide therapy. Tirzepatide promotes weight loss and improves glycemic control mainly through incretin-mediated effects on appetite regulation, food intake, gastric emptying, insulin secretion, and body weight (Thiriveedi, 2025; Hossain et al., 2025; Rochira et al., 2024). However, lifestyle modification, especially increased physical activity and reduced sedentary behavior, may independently improve microbiota diversity, enhance the abundance of metabolically favorable taxa, support intestinal barrier function, and reduce inflammatory tone (Strasser et al., 2021; Muralidharan et al., 2021; Wegierska et al., 2022). Therefore, in individuals with obesity treated with tirzepatide, changes in gut microbiota composition should be interpreted in the context of concurrent lifestyle factors. Physical activity may complement tirzepatide-induced metabolic improvement by contributing to microbial and host metabolic adaptations, whereas uncontrolled variation in lifestyle habits may confound the attribution of microbiota changes specifically to tirzepatide.

### 4.2 Diet and dietary bioactives

Diet is one of the strongest modulators of gut microbiota composition and metabolic activity. Carbohydrate- and fiber-rich diets provide fermentable substrates that support microbial production of short-chain fatty acids, whereas low-carbohydrate or low-fiber diets may reduce butyrate-producing bacteria such as *Bifidobacterium, Roseburia*, and *Eubacterium rectale*, potentially affecting colonocyte energy supply, intestinal permeability, and inflammation (Zambrano et al., 2024a; Cadena-Ullauri et al., 2025; Xiong et al., 2022).

High-fiber dietary patterns can favor acetate- and propionate-producing taxa, including members of Bacteroidetes and Actinobacteria, whereas high-fat dietary patterns may promote dysbiosis, increase exposure to bacterial lipopolysaccharide, and contribute to inflammatory and barrier-related alterations (Zambrano et al., 2024a; Cadena-Ullauri et al., 2025; Xiong et al., 2022; Ley et al., 2005; Lopez-Legarrea et al., 2014; Ye et al., 2024). In addition, polyphenol-rich foods, particularly fruits and vegetables, may favor beneficial genera such as *Bifidobacterium* and *Lactobacillus* and modulate host pathways related to lipid metabolism (Bagheri et al., 2022).

Specific dietary bioactives may further influence microbial composition. Prebiotics such as fructooligosaccharides can promote beneficial bacterial populations, including bifidobacteria. Catechins may reduce the abundance of bacterial groups such as *Bacteroides* spp., *Clostridium* spp., and *Escherichia coli*, while anthocyanins from red fruits have been reported to inhibit potential pathogens such as *Helicobacter pylori* and *Staphylococcus* spp. (Carrera-Quintanar et al., 2018). These findings support the relevance of diet as a microbiota-modulating strategy, although the magnitude and direction of these effects depend on dietary pattern, food matrix, baseline microbiota composition, and host metabolic status.

Dietary intake is also particularly relevant when interpreting microbiota changes during tirzepatide therapy. Tirzepatide reduces appetite, delays gastric emptying, and decreases energy intake, which may alter the quantity and type of substrates reaching the intestinal microbiota (Wang et al., 2025; Ma et al., 2025; Thiriveedi, 2025; Hamza et al., 2025). Consequently, treatment-associated changes in microbial composition, SCFA production, bile acid metabolism, or inflammatory signaling may partly reflect shifts in dietary intake rather than direct pharmacological modulation of the microbiota (Wang et al., 2025; Ma et al., 2025). For this reason, studies evaluating tirzepatide and the gut microbiota should carefully document dietary patterns, fiber intake, fat intake, caloric restriction, and use of prebiotics, probiotics, or bioactive-rich foods. Integrating dietary assessment with microbiome and metabolomic profiling would help distinguish drug-specific microbial effects from diet-mediated adaptations during weight loss.

### 4.3 Host genetics and interindividual variability

Host genetic background may partly contribute to interindividual variability in gut microbiota composition, microbial metabolism, and obesity-related metabolic phenotypes. Although environmental factors such as diet, lifestyle, medication exposure, and metabolic status are major determinants of the gut microbiota, host genetic variation can influence intestinal physiology, immune regulation, bile acid metabolism, epithelial barrier integrity, lipid handling, and inflammatory responses, thereby shaping the ecological conditions in which microbial communities develop (Wu et al., 2023, 2021; Ha et al., 2024; Li et al., 2022; He et al., 2021).

Several host genes and genetic variants have been associated with differences in microbial taxa involved in metabolic regulation. For example, the rs738409 variant in *PNPLA3*, which has been linked to hepatic fat accumulation and metabolic dysfunction-associated steatotic liver disease, has also been associated with changes in taxa involved in fatty acid metabolism, including Bacteroidetes, *Gemmiger*, and *Oscillospira* (Kravetz et al., 2020). Similarly, differences in *GPR35* gene expression have been associated with increased abundance of *Ruminococcus gnavus* and hepatic fat accumulation, suggesting a potential link between host receptor signaling, gut microbial ecology, and liver metabolic phenotypes (Wu et al., 2023).

Other host-regulated pathways may also influence microbial communities relevant to obesity. Intestinal *HIF-2*α signaling dysregulation has been associated with increased *Bacteroides vulgatus* and reduced *Ruminococcus torques*, taxa implicated in thermogenesis, lactate metabolism, and bile acid-related metabolic regulation (Wu et al., 2021). Similarly, disruption in *SQLE* gene expression has been associated with changes in taxa related to cholesterol metabolism and intestinal barrier integrity, including *Desulfovibrio fairfieldensis* and members of Lachnospiraceae (Ha et al., 2024; Li et al., 2022). Additional variants, including rs6065904 in *PTLP*, rs9363741 in *AL365503.1*, rs148330122 in *SIPA1L3*, and rs3010562 in *TTLL2*, have been linked to changes in bacterial groups such as *Erysipelotrichaceae, Lachnobacterium, Bacilli*, and *Anaerofilum* (He et al., 2021).

These findings suggest that host genetics may influence microbiota composition and function through pathways related to lipid metabolism, bile acid signaling, immune tone, and intestinal barrier regulation. However, most reported associations remain observational and require functional validation. Therefore, genetic effects should be interpreted as contributors to interindividual variability rather than deterministic drivers of obesity-associated dysbiosis.

This variability is particularly relevant when evaluating microbiota changes during tirzepatide therapy. Tirzepatide improves body weight, glycemic control, insulin sensitivity, and adiposity through dual GIP and GLP-1 receptor agonism (Thiriveedi, 2025; Hamza et al., 2025), but the magnitude of clinical and microbial responses may vary among individuals. Host genetic background may influence this heterogeneity by shaping baseline microbiota composition, microbial metabolite production, bile acid metabolism, inflammatory tone, or enteroendocrine signaling (Goodrich et al., 2014). However, current evidence does not establish a direct relationship between specific host genetic variants and microbiota-mediated responses to tirzepatide. Future studies should therefore integrate host genotyping, longitudinal microbiome profiling, metabolomics, dietary assessment, and metabolic phenotyping to determine whether genetic background contributes to differential microbiota adaptation or therapeutic response during tirzepatide treatment.

### 4.4 Medication exposure as a microbiota-modifying factor

Medication exposure is an important but often underrecognized modulator of the gut microbiota. In obesity studies, medication use should be considered both as a confounder and as a potential therapeutic modifier of microbial ecology (Baldanzi et al., 2026; Suvvari et al., 2025). In the context of individuals with obesity, patients are frequently receiving multiple pharmacological interventions for associated conditions, including type 2 diabetes mellitus, hypertension, dyslipidemia, gastroesophageal reflux disease, and recurrent infections. Therefore, the microbiota profiles in this population may reflect not only obesity-related dysbiosis, but also the cumulative effects of medication exposure.

Antibiotics represent one of the most extensively studied medication classes affecting the gut microbiota (Patangia et al., 2022; Baldanzi et al., 2026; Li et al., 2025). Amoxicillin, amoxicillin–clavulanic acid, vancomycin, ciprofloxacin, clindamycin, and broad-spectrum antibiotics have been associated with shifts in bacterial composition, including increased abundance of opportunistic taxa such as *Escherichia, Shigella, Klebsiella, Parabacteroides*, and *Enterobacter*. These changes may occur together with reductions in beneficial anaerobic or SCFA-producing taxa such as *Faecalibacterium, Ruminococcus, Roseburia, Lachnospira, Coprococcus*, and *Dorea* (Mamieva et al., 2022; Pallav et al., 2014; Kabbani et al., 2016; Rashid et al., 2015; Burdet et al., 2019). Functionally, these alterations may reduce microbial diversity, impair short-chain fatty acid production, disrupt bile acid metabolism, compromise epithelial barrier integrity, and increase susceptibility to inflammatory signaling (Hu et al., 2017).

Beyond antibiotics, several drugs commonly used in individuals with obesity may also influence microbial ecology and metabolic outputs (Gofron et al., 2025; Vich Vila et al., 2020). Antidiabetic agents, lipid-lowering drugs, proton pump inhibitors, antihypertensive medications, and anti-inflammatory therapies can modify microbial composition, metabolite production, intestinal pH, bile acid availability, gut motility, or inflammatory tone (Gofron et al., 2025; Vich Vila et al., 2020; Zawistowska-Rojek and Tyski, 2025). These effects are relevant because they may overlap with, mask, or amplify microbiota changes attributed to weight loss or anti-obesity therapies. Consequently, medication exposure should be carefully documented in studies evaluating obesity, gut microbiota, microbial metabolites, and response to metabolic interventions.

Tirzepatide may influence the gut microbiota indirectly through reduced appetite, decreased caloric intake, delayed gastric emptying, improved glycemic control, changes in bile acid metabolism, and reduced inflammatory burden (Wang et al., 2025; Ma et al., 2025). However, microbiota changes observed during tirzepatide treatment should not be interpreted as drug-specific effects unless medication history is controlled. Future tirzepatide–microbiota studies should systematically record baseline and follow-up medication exposure, antibiotic use within defined time windows, concomitant antidiabetic therapies, and other drugs known to affect microbial composition or host metabolism.

## 5 Tirzepatide and the gut microbiota–obesity axis: evidence and potential mechanisms

Tirzepatide is a dual glucose-dependent insulinotropic polypeptide receptor and glucagon-like peptide-1 receptor agonist used for the management of obesity and metabolic disease (Wiechert and Holzapfel, 2022; Zambrano et al., 2024b; Corrao et al., 2024). Its metabolic effects include improved glucose-dependent insulin secretion, reduced appetite, delayed gastric emptying, enhanced glycemic control, weight reduction, and improvements in lipid metabolism and energy balance (Corrao et al., 2024). Given the close relationship between host metabolism and the intestinal ecosystem, attention has been directed toward whether tirzepatide may also influence gut microbiota composition and function in obesity.

### 5.1 Tirzepatide-associated microbiota changes

Current evidence suggests that tirzepatide treatment may be associated with changes in microbial taxa involved in intestinal barrier integrity, inflammatory regulation, and metabolic homeostasis. For example, increases in genera such as *Akkermansia* and *Romboutsia* have been reported after treatment, both of which have been associated with mucosal barrier function and reduced inflammatory signaling (Ma et al., 2025). These findings are biologically relevant because obesity-associated dysbiosis can impair epithelial barrier integrity, increase exposure to microbial components such as LPS, and activate inflammatory pathways including TLR4/NF-κB signaling (Ye et al., 2024; Ma et al., 2025; Bailey et al., 2025).

Tirzepatide may also influence the abundance of taxa linked to inflammatory and metabolic dysfunction. Reductions in bacteria such as *Erysipelatoclostridium, Bacteroides*, and *Lachnoclostridium* have been reported in association with tirzepatide treatment (Ma et al., 2025). A reduction in these bacteria could contribute to lower LPS-mediated signaling, improved insulin sensitivity, and better lipid metabolism. Mechanistically, reduced activation of TLR4-related inflammatory pathways may limit inhibitory serine phosphorylation of insulin receptor substrate-1, thereby supporting insulin signaling and reducing downstream metabolic disturbances associated with insulin resistance, hyperglycemia, and excessive triglyceride accumulation (Ma et al., 2025; Alshehri et al., 2025).

### 5.2 Potential mechanisms linking tirzepatide, microbiota, and metabolic improvement

The potential interaction between tirzepatide and the gut microbiota may involve several mechanisms described in the microbiota–obesity axis. First, tirzepatide-related improvements in glycemic control and adiposity may reduce systemic inflammatory tone, which could indirectly favor a more stable intestinal microbial ecosystem (Thiriveedi, 2025; Hossain et al., 2025; Rochira et al., 2024). Second, changes in appetite, food intake, gastric emptying, and nutrient availability may alter the intestinal environment and microbial substrate use (Cortés-Martín et al., 2020; Rinninella et al., 2019; Zambrano et al., 2023). Third, microbiota-associated pathways involving SCFA production, bile acid metabolism, epithelial barrier function, and inflammatory signaling may contribute to metabolic adaptation during treatment (Wang et al., 2025; Moles and Otaegui, 2020).

These pathways provide a biological framework for interpreting microbiota changes during tirzepatide therapy. However, current evidence does not establish whether microbial shifts actively mediate treatment response or mainly reflect reduced food intake, weight loss, altered gastrointestinal motility, and improved glycemic control (Wang et al., 2025; Corrao et al., 2024; Ma et al., 2025; Alshehri et al., 2025; Moles and Otaegui, 2020).

Importantly, tirzepatide should not be interpreted solely as a direct microbiota-modifying agent. Rather, its effects may reshape the intestinal ecosystem through host-mediated changes in energy intake, nutrient delivery, gastrointestinal transit, glycemic control, bile acid signaling, and inflammatory status. In this framework, the gut microbiota may function both as a responder to tirzepatide-induced metabolic improvement and as a potential contributor to downstream changes in barrier integrity, microbial metabolite production, and immune-metabolic regulation.

### 5.3 *Lachnospiraceae*, microbial metabolites, and extra-intestinal effects

Some studies suggest that tirzepatide may affect members of the family Lachnospiraceae, a taxonomically and functionally diverse group involved in carbohydrate fermentation and SCFA production (Chen et al., 2025). These changes may be relevant because Lachnospiraceae-derived metabolites can participate in intestinal and systemic metabolic regulation (Vacca et al., 2020). However, this family includes taxa with different and sometimes opposing functions; therefore, changes in Lachnospiraceae should not be interpreted as uniformly beneficial or harmful without species-, strain-, and function-level resolution (Chen et al., 2025).

The reported association between tirzepatide, microbial metabolites, and bone-related outcomes also requires cautious interpretation. Although microbial metabolite changes may influence extra-intestinal processes, including bone metabolism, current evidence remains limited and appears to be largely preclinical. Therefore, potential adverse or beneficial microbiota-related effects of tirzepatide should be supported by direct testing before being translated into clinical conclusions.

### 5.4 Direct vs. indirect microbiota effects

A central unresolved question is whether tirzepatide directly modifies the gut microbiota or whether observed microbial changes are secondary to metabolic improvement. Weight loss, reduced caloric intake, delayed gastric emptying, improved glycemic control, altered bile acid metabolism, changes in gastrointestinal motility, and dietary modifications during treatment may all influence microbiota composition (Ma et al., 2025; Wang et al., 2025; Chen et al., 2025). Therefore, tirzepatide-associated microbiota changes should be interpreted as part of a bidirectional host–microbiota adaptation rather than as a simple direct pharmacological effect.

Clarifying this distinction will require longitudinal human studies with baseline and follow-up microbiota profiling, detailed dietary assessment, metabolic phenotyping, inflammatory markers, bile acid and SCFA measurements, and functional microbiome analysis. Such studies will be essential to determine whether microbiota signatures can predict tirzepatide response, whether microbiota modulation contributes to treatment efficacy, and whether microbiota-targeted strategies could complement incretin-based obesity therapy.

## 6 Future perspectives

Future studies should determine whether baseline microbiota composition or microbial metabolite profiles predict clinical response to tirzepatide. Longitudinal designs should collect microbiota, diet, medication exposure, inflammatory markers, bile acids, SCFAs, glycemic parameters, and anthropometric outcomes before and during treatment. This approach would help distinguish direct drug-associated microbial effects from changes secondary to weight loss, dietary modification, delayed gastric emptying, or improved glycemic control. Multi-omics approaches, including metagenomics, metabolomics, bile acid profiling, transcriptomics, and immune phenotyping, will be essential to identify microbial pathways associated with improved adiposity, inflammation, and therapeutic response. Controlled trials should also assess whether microbiota-directed interventions, including fiber, prebiotics, probiotics, synbiotics, polyphenol-rich diets, and structured lifestyle programs, can enhance tirzepatide efficacy or support long-term weight maintenance.

## 7 Conclusion

The gut microbiota contributes to obesity-related metabolic dysfunction through microbial metabolite production, intestinal barrier regulation, immune activation, endocrine signaling, and gut–brain communication. Dysbiosis may promote impaired energy balance, insulin resistance, adipose tissue dysfunction, and chronic low-grade inflammation, although these relationships are context-dependent and cannot be attributed to a single bacterial taxon or metabolite. Tirzepatide has shown efficacy in obesity and metabolic disease through dual GIP and GLP-1 receptor agonism, improving appetite regulation, glycemic control, body weight, and lipid metabolism. Evidence suggests that tirzepatide may also be associated with changes in gut microbiota composition and function, particularly in taxa and metabolites related to barrier integrity, SCFA production, inflammation, and metabolic homeostasis.

However, it remains unclear whether these microbiota changes are direct pharmacological effects or secondary consequences of weight loss, reduced caloric intake, delayed gastric emptying, improved glucose control, or dietary modification. Longitudinal multi-omics studies in diverse populations are therefore needed to determine whether microbiota signatures can predict tirzepatide response and whether microbiota-targeted strategies can complement pharmacological treatment for obesity.
